# Effect of Dietary Administration of Green Macroalgae (*Ulva intestinalis*) on Mucosal and Systemic Immune Parameters, Antioxidant Defence, and Related Gene Expression in Zebrafish (*Danio rerio*)

**DOI:** 10.1155/2022/7693468

**Published:** 2022-11-04

**Authors:** Elaheh Rouhani, Roghieh Safari, Mohammad Reza Imanpour, Seyed Hossein Hoseinifar, Metin Yazici, Ehab El-Haroun

**Affiliations:** ^1^Department of Fisheries, Faculty of Fisheries and Environmental Sciences, Gorgan University of Agricultural Sciences and Natural Resources, Gorgan, Iran; ^2^Iskenderun Technical University, Faculty of Marine Sciences and Technology, Iskenderun, Hatay, Turkey; ^3^Fish Nutrition Research Laboratory, Animal Production Department Faculty of Agriculture Cairo University, Cairo, Egypt

## Abstract

This study investigated the effects of adding green macroalgae gutweed (*Ulva intestinalis*) powder to zebrafish (*Danio rerio*) feed at different levels on innate immune responses, antioxidant defence, and gene expression. A total of 600 zebrafish (0.3 ± 0.08 g) were randomly allocated to 12 aquariums in four treatments with three replicates (50 fish per aquarium). Zebrafish were fed with different levels of *U. intestinalis* powder 0, 0.25, 0.5, and 1% for eight weeks. Whole-body extract (WBE) immune parameters including total protein level, globulin level, and lysozyme activity were evaluated and revealed statistically significant increased in all *U. intestinalis* supplemented groups compared to the control (*P* < 0.05). However, mucus immune parameters (total protein, globulin, and lysozyme) were statistically different in only 1% gutweed supplemented groups from other groups. While glutathione peroxidase (GPx) and superoxide dismutase (SOD) increased with the addition of gutweed (*P* < 0.05), catalase (CAT) did not change (*P* > 0.05). The study results showed that dietary gutweed remarkably upregulated immune-related genes such as lysozyme (Lyz) and Interleukin 1 beta (IL-1*β*). Antioxidant-related genes (SOD and CAT) and growth-related genes, including growth hormone (GH) and insulin-like growth factor-I (IGF-1), were remarkably upregulated with gutweed treatment (*P* < 0.05). In conclusion, dietary *U. intestinalis* showed beneficial effects on immunity, and same effects were observed in case of antioxidant and growth related genes expression in zebrafish.

## 1. Introduction

Studies on the determination of alternative feed additives for the aquaculture sector are increasing. The probiotics [1], prebiotics, synbiotics, organic acid [2], and medicinal herb [3] are among the alternative feed additives which showed beneficial effects in aquaculture [4, 5]. Seaweed or seaweed-derived ingredients recieved increasing attention as alternative feed additives in fish feed formulations [6, 7], as they are highly nutritious [8] and have exceptionally versatile properties [9]. Seaweeds (macroalgae) have widely been used as functional feed additives to improve fish growth [10, 11], nutrient utilisation, stress response [11], fish health, and disease resistance [12] as well as skin pigmentation in ornamental fish [11].

Recently, macroalgae and their extracts have received worldwide attention due to their bioactive compounds [10, 13], including polysaccharides [14], proteins, polyunsaturated fatty acids [15], pigments, polyphenols, minerals, vitamins, and plant growth hormones [16]. However, seaweed components may vary considerably depending on the species [17], time of harvesting, habitat, and external conditions including water temperature, nutrient concentration, and light intensity in the water [9].

The green macroalgae (Chlorophyta) comprises a minor part (<1%) of total algae production ([18, 19]. However, they are rich in beneficial compounds such as polysaccharides, particularly sulfated proteins, amino acids, chlorophylls and carotenoids, polyunsaturated fatty acids, minerals, and vitamins with medicinal and health-promoting effects [13, 20]. *U.intestinalis*, known as gutweed and grass kelp, is found naturally throughout the world [21, 22]. It has been reported that *Ulva* sp. and its derivatives such as ulvan had immunostimulatory effects and increased disease resistance in various fish and shrimp species [23], including gilthead sea bream (*Sparus aurata*), European sea bass (*Dicentrarchus labrax*) [6], Pacific white shrimp (*Litopenaeus vannamei*) [10], and banana shrimp (*Fenneropenaeus merguiensis*) [24] diets. On the other hand, different studies have shown that the extracts derived from plants, seaweed, and microalgae can neutralise free radicals in fish tissues and, therefore, can be used as natural sources of antioxidants [11].

Zebrafish (*Danio rerio*) has been used as a suitable model fish in feeding and immunity studies due to their short life cycle, low cost, easy manipulation, and physiological similarity to most farm species [25, 26]. Therefore, research in zebrafish nutrition can allow essential contributions in many areas of research for finfish aquaculture [27, 28].

Previous studies revealed that some green macroalgae may be used as feed additives in fish [7] and crustacean diets [10]. However, in these studies, the effects of *U. intestinalis* on skin mucus immunity have not been investigated. Morever, as far as we know, the effects of “U. intestinalis” at the molecular level on growth, antioxidant, and immunity have not been explored in fish. Therefore, a holistic approach was exhibited in this study, and the effects of *U. intestinalis* on skin mucus immunity, nonspecific immunity, and antioxidant enzyme activities were investigated, as well as the effects of growth, antioxidant response, and immune defence were examined at the molecular level in fish.

## 2. Materials and Methods

### 2.1. Preparing of *U. Intestinalis* for Diet

*U. intestinalis* specimens were handpicked from the shores of Iskenderun Bay at a depth of 0-20 m Iskenderun, Hatay, Turkey (36.26.49 N 35.52.24 E). The collected macroalgae were prepared for use by washing, drying, and storing in cooler containers at the Iskenderun Technical University Algal Biotechnology Laboratory [29]. *U. intestinalis* was first washed thoroughly with ambient water to remove foreign substances such as sand and adhering substances and brought to the laboratory environment in sterile polyethylene bags. To remove epiphytic organisms and necrotic particles on the samples, they were washed with distilled water in the Algal Biotechnology Laboratory. A shaded area in the laboratory environment that was not exposed to the sun for the drying process was used. Identification studies of macroalgae were carried out in Algal Biotechnology Laboratory based on the schemes reported in the literature and using the Ckx41sf model stereo inverted light microscope from Olympus. The macroalgae, which were ground with a properly dried homogeniser, were stored at +4°C until they were added to the feed [30, 31].

### 2.2. Preparation of Diets and Experimental Design

Table 1 indicates the proximate analysis of the basal diet, a commercial diet (BioMar SAS, Nersac, France). The experimental diets were formed by adding the crude *U. intestinalis* powders, which were ground to obtain fine powders, to the control diet at a ratio of 0, 0.25, 0.5, and 1%, as described earlier [17]. In the process of preparing the experimental diets, the basal diet was pulverized, and algae powders were added to correspond diet at the expense of cellulose (to equalize the total energy content). Then, it was mixed thoroughly by means of a blender; after that, water was added to form a mixture. Finally, it was repelleted via a meat grinder, air-dried, ground, and sieved to obtain a suitable size (1 mm). The prepared diets were stored at 4°C in sealed and labelled packages until the study was conducted.

### 2.3. Fish Rearing Conditions and Feeding

600 six-week-old zebrafish used in the study were procured from Ornamental Fish farm (Golestan Province, Iran) and transported in polyethylene bags with oxygen-filled water to the GUASNR (Gorgan University of Agricultural Sciences and Natural Resources) Zebra Lab. Fish were adapted for two weeks. During this period, fish were fed with a commercial diet (Biomar, France) three times a day. Then, zebrafish with approximate weight of 0.3 ± 0.08 g were randomly assigned into triplicate groups as follows: 0 (control; C), 0.25%, and 0.5% and 1% (each group contains 50 fish) in 100 L aquarium (half filled with water, 50 L) During the lasting eight weeks of the experiment, the fish were fed the experimental diets as *ad libitum* three times daily until they reached apparent satiation. In the study, in which the static culture system was used, continuous ventilation was provided using an air stone. Approximately 50% of the total water in each aquarium was changed every two days to maintain water quality. During the trial, water quality parameters were measured regularly using a portable device (WTW, Munich, Germany); water temperature, pH, and dissolved oxygen were as follows, respectively, 24.0 ± 0.8°C, 7 ± 0.1, and 7.6 ± 0.2 mg L^−1^. The current study was carried out under the protocol approved by the committee of ethics of the faculty of sciences of the University of Tehran (357; 8 November 2000).All procedures were performed in compliance with relevant laws and institutional guidelines.

### 2.4. Skin Mucus and Whole-Body Extract (WBE) Nonspecific Immune Responses

#### 2.4.1. Sampling for Immunologic Parameters

As the fish were too small to collect blood, the protocol described in our previous study, Yousefi et al. [32], which was based on Holbech et al. [33], was followed to obtain WBE of nine fish per each treatment (0, 0.25, 0.5, and 1% with *U. intestinalis*). Briefly, the heads and fins were cut. Then, by using a mortar filled with liquid nitrogen, the samples were crushed, and twice the tissue weight of homogenate, buffer was added as described by Holbech et al. [33]. The homogenate was centrifuged, and the supernatant was stored -80°C until used.

At the end of the experiment, mucus sampling was also done, as Hoseinifar et al. [34] stated. Nine 24 hours fasted fish were randomly selected from each treatment group (three samples per replicate; each replicate as a separate data). Fish exposed to 500 g L^−1^ of clove powder were transferred to a polyethylene bag containing 5 mL of 50 mM NaCl (Sigma, Steinheim, Germany) for 2 minutes. During this period, the fish were smoothly rubbed inside the zip bags. The obtained mucus samples were quickly transferred to 15 ml sterile tubes and centrifuged at 1500 g for 10 minutes at 4°C. Supernatants were kept at -80°C until used [35].

#### 2.4.2. Assessment of Nonspecific Immune Response

This study measured lysozyme activity as well as levels of total protein and globulin to assess nonspecific immune parameters in mucus and WBE in the treatment groups. Total protein level in mucus samples and WBE was determined according to Lowry et al. [36] and Bradford [37], 2011's protocol. The method described by Guardiola et al. [38] was used to measure lysozyme activity in both skin mucus and WBE in zebrafish fed experimental diets, with some modifications. Briefly, equal volumes (100 *μ*l) of mucus or WBE samples were mixed with the bacterial suspension of *Micrococcus luteus* (Sigma) and incubated at 30°C. The resulting decrease in optical density at 450 nm (OD450) was measured using a microplate reader (Benchmark, BioRad, USA) at 25°C for 15 minutes. The amount of sample that causes an absorbance decrease of 0.001 per minute is defined as one unit of lysozyme activity. The method described by [39] was used to determine the total globulin level of skin mucus and WBE. Briefly, total protein levels were measured both before and after precipitation of Ig molecules using a 12% polyethylene glycol (Sigma) solution. The difference in protein content was considered as the globulin content of skin mucus and WBE.

### 2.5. Antioxidant Defence

The WBE catalase (CAT) activity was measured using a commercially available kit (ZellBio GmbH, Germany) following the instruction of the manufacturer, as we described in our previous paper [40]. As suggested by ([41]), a commercial kit (Zellbio®, Berlin, Germany) was employed to determine the WBE glutathione peroxidase (GPx) as well as superoxide dismutase (SOD).

### 2.6. Gene Expressions

#### 2.6.1. Sampling

After 8 weeks of feeding study, nine zebrafish were randomly sampled from each aquarium (replicate) and anesthetized using 500 mg L^−1^ clove solution. Samples from the whole fish brain, liver, and intestinal tissues were rapidly removed. Samples from each replicate pooled [42] and held in liquid nitrogen in a deep freezer (-80°C) until further analysis.

#### 2.6.2. RNA Extraction and cDNA Synthesis

Extraction of total RNA of samples from 100 mg tissue was done using Esterabad-Zistfan-Pishro-Azma. Then, the isolated RNA was treated with DNase I (Fermentas, Lithuania) to remove genomic DNA. NanoDrop (Nanodrop Technology, Wilmington, DE, USA) and 1.5% Agarose gel were used to control the RNA concentration and quality in each sample. The transcription of total RNA (5 *μ*l with approximate concentration of 500 ng) to cDNA was performed using the cDNA synthesis kit (Fermentas, Lithuania) in accordance with the company's recommended protocol [42].

#### 2.6.3. Real-Time PCR

The primers used to detect immunity, antioxidant, and growth-related gene expression were shown in Table 2. Primers were designed in accordance with both GenBank sequences and Primer3 software as described by Safari et al. [35]. Quantitative real-time PCR (qPCR) assays were performed to study alteration in the expression of immune (IL-1*β* and lysozyme-C), antioxidant (SOD and CAT), and growth-related genes (GH and IGF-1). The expression of immune-related genes was measured in intestine, antioxidant, and IGF-1 in liver and GH in brain. The relative real-time PCR was fulfilled using an iCycler (BioRad, USA) and a SYBR Green qPCR Master Mix (Fermentase, Lithuania) as explained in our previous paper [43].

### 2.7. Statistical Analysis

The Pfaffl formula [44] was used to calculate relative gene expression. Analysis of the ratio between targets and housekeeping (*β*-actin) genes was performed with REST software. Using the Kolmogorov-Smirnov test, the normality of the distributions of both immune parameters and gene expression data was evaluated. The obtained data were subjected to one-way ANOVA with *α* = 0.05 and then Duncan's test. Data were reported as mean ± standard deviation (*X* ± S.D). Statistical analyses were performed with SPSS 19 software (SPSS, USA).

## 3. Results

### 3.1. Immune Response

Table 3 represents nonspecific immune system parameters in skin mucus and WBE of zebrafish after an 8-week feeding trial. While the amount of mucosal total protein and mucosal globulin showed slight increases in the 0.25 and 0.5 supplemented groups, which were not statistically significant compared to control, and the increases in the 1% supplemented group were statistically significant (*P* < 0.05). The lysozyme activity in skin mucus of 1% *U. intestinalis* fed fish was significantly higher than other treatments (*P* < 0.05).

Immunological parameters, such as the level of total protein, globulin level, and lysozyme activity in the WBE, showed statistically significant increases in all *U. intestinalis* supplemented groups compared to the control group (*P* < 0.05). No statistical difference was observed among the groups with *U. intestinalis* supplementation (*P* > 0.05).

### 3.2. Effects on Antioxidant Enzyme Activities

The effects on antioxidant activity such as SOD, CAT, and GPx in zebrafish fed with *U. intestinalis* feed were evaluated, and the results are shown in Table 4. SOD exhibited significantly higher activity in 0.5% and 1% *U. intestinalis* supplemented groups than control and 0.25% supplemented groups with the highest level in 1% supplemented group (*P* < 0.05). Similarly, the activity of GPx in fish fed 1% *U. intestinalis* diets was higher than the other treatments (*P* < 0.05). However, no significant difference was found in CAT activity (*P* > 0.05).

### 3.3. Gene Expressions

**Figure 1:**
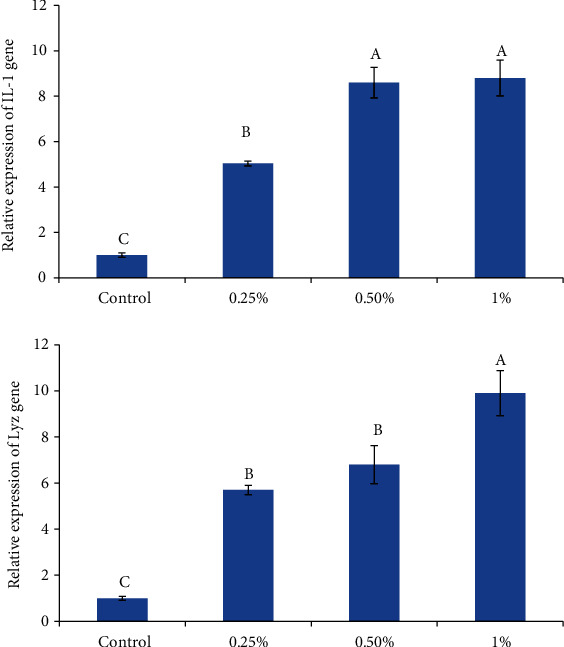
The effects of different levels of *U. intestinalis* on immune-related gene expression in the intestine of zebrafish (*n* = 9). Values are presented as the mean ± S.D. The bars assigned with different letter denote significant difference between treatments (*P* < 0.05).

**Figure 2:**
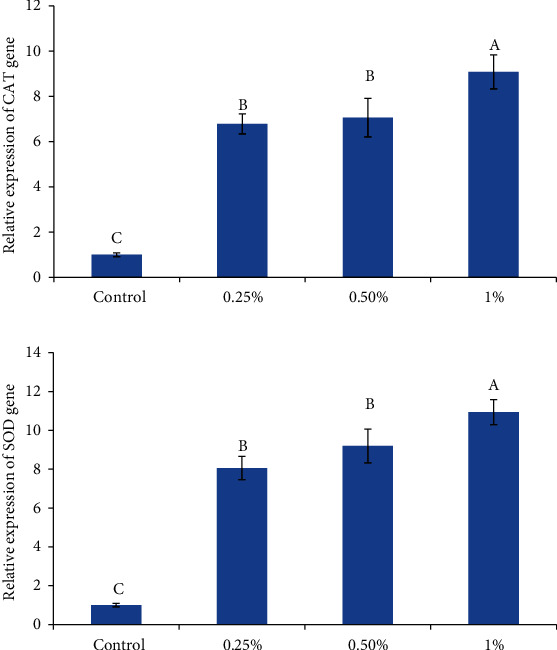
The effects of different levels of *U. intestinalis* on antioxidant enzyme gene expression in the intestine of zebrafish. Values are presented as the mean ± S.D. The bars assigned with different letter denote significant difference between treatments (*P* < 0.05).

**Figure 3:**
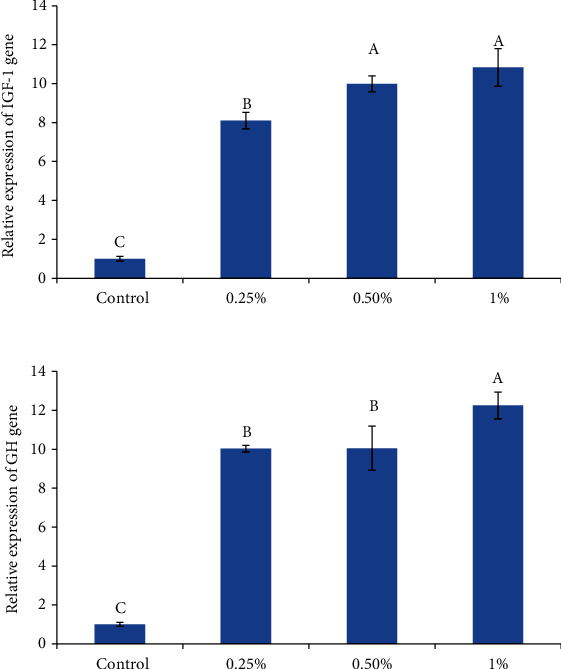
The effects of different levels of *U. intestinalis* on GH in brain and IGF-1 in liver of zebrafish (*n* = 9). Values are presented as the mean ± S.D. The bars assigned with different letter denote significant difference between treatments (*P* < 0.05).

The effects on the expression of immune-related genes (Lyz and IL-1*β*), growth-related genes (GH and IGF-1), and antioxidant-related genes (SOD and CAT) of zebrafish which fed diets supplemented with *U. intestinalis* powder are shown in Figures 1–3, respectively.

Expression of Lyz and IL-1*β* genes showed significant differences in all *U. intestinalis* containing groups in related to the control group (Figure 1). Expression of IL-1*β* was significantly higher than that in fish fed 0.5 and 1% *U. intestinalis* compared control and 0.25% *U. intestinalis* groups (*P* ≤ 0.05). As for Lyz gene expression, the highest increase was obtained in the group with 1% addition, and no difference was detected between the groups with 0.25% and 0.5% additions. The expression of the IL-1*β* gene was 5.04, 8.6, and 8.8, and Lyz gene was 5.7, 6.8, and 9.9 fold of control in *U.intestinalis* treated groups (0.25%, 0.5%, and 1%), respectively, which showed dose-dependent reduction pattern (*P* < 0.05) (Figure 1).

Both antioxidant-related genes (SOD and CAT) were remarkably upregulated with *U. intestinalis* supplementation with respect to control (Figure 2). While the highest expressions' levels in SOD and CAT genes were observed in the 1% *U. intestinalis* supplemented group, no difference was observed in the 0.25% and 0.5% supplemented groups. A dose-dependent upward pattern was observed in the expression of SOD and CAT. The expression of the SOD gene was 8.6, 9.4, and 10.94, and CAT gene was 6.76, 7.06, and 9.8 fold of control in *U.intestinalis* treated groups (0.25%, 0.5%, and 1%) (Figure 2).

Similar to antioxidant and immune-related genes, growth-related genes (GH and IGF-1) were also upregulated in fish fed to all *U. intestinalis* containing diets compared to the control group (Figure 3). The expression of the GH gene was 10.03, 10.5, and 12.25, and IGF-1 gene was 8.01, 9.9, and 10.83 fold of control in *U.intestinalis* treated groups (0.25%, 0.5%, and 1%), respectively. There was no difference in GH expressions between 0.25% and 0.5% *U. intestinalis* supplemented groups, and the highest expression level was observed with 1% *U. intestinalis*.

## 4. Discussions

Macroalgae has received more attention due to its rich source of bioactive compounds [9]. Macroalgae and/or their extracts contribute to the improvement of the health status of aquatic animals and increase productivity, offering a great potential to the aquaculture sector, and providing healthy food for consumers [45]. In addition, it has been shown that macroalgae and some products obtained from macroalgae can improve some parameters of innate immune response such as serum lysozyme [46], alternative complement pathway [15], and phagocytic activity in cultured fish [47] and crustaceans [10, 24]. Although the exact mechanism is not known, it has been suggested that the effect of macroalgae to increase mucosal immunity may be more than systemic immunity [6]. Several studies have suggested that macroalgae and their extracts can be used as safer prophylactic and therapeutic alternatives to antibiotics in the control of infectious diseases affecting farmed fish [45] due to their strong antiviral [10] and antibacterial properties [48] against virus and some bacterial fish pathogens [9, 49].

Lysozyme, which has lytic activity against both gram-positive and gram-negative bacteria, has been proven to be involved in a wide variety of protective mechanisms such as activate the complement system and phagocytes and can be found in the mucus, lymphoid tissue, plasma, and other body fluids of fish. Therefore, lysozyme is a very important factor in determining the innate immunity of fish [15, 50]. In this study, feeding zebrafish with diets containing *U. intestinalis* powders showed a remarkable effect on lysozyme activity. In line with our work, Akbary and Aminikhoei [46] reported that the lysozyme activity of mullet fish fed 1% supplemented water-soluble extract of *Ulva rigida* was remarkably higher than that of fish fed the control diet and rest of other treatment levels. In another study, sea bass fed with 2.5% *U. intestinalis* diet showed significantly higher lysozyme activity than those fed with control and 7.5% *U. intestinalis* added diets [51]. Since the skin mucus of fish contains many different biologically active molecules, it plays a vital role in preventing the entry of pathogens into the body and in immunity [50]. Martinez-Antequera et al. [6] reported that the inclusion of *Ulva onhoi* in feed resulted in an increase in skin mucus lysozyme activity and alkaline phosphatase in sea bream and sea bass. Additionally, Vazirzadeh et al. [52] stated that rainbow trout fed diets containing different marine algae such as 5% and 10% of *Gracilariopsis persica*, 5% and 10% of *Hypnea flagelliformis*, and 5% of *Sargassum boveanum* and showed high lysozyme activity similar to the results in our study. Moreover, Liu et al. [24] suggested that Enteromorpha polysaccharides application can be used to support the shrimp immune system as it leads to an increase in serum phenoloxidase levels, lysozyme activity, and phagocytic activity in banana shrimps (*Fenneropenaeus merguiensis*).

Total proteins are generally considered as a clinical indicator of many conditions in fish, such as health, stress, and nutritional status [53]. In the present study, the amount of total protein in WBE and skin mucus increased in fish groups fed a 1% supplement diet. In agreement with our findings, Harikrishnan et al. [49] reported that total protein amount increased in both *L. rohita* fed all ulvan supplemented diets challenged with *F. columnaris* and *L. rohita* fed ulvan supplemented diets unchallenged compared to control group. However, [54] suggested that the serum total protein level of tilapia is not affected by dietary levels of the green alga *Ulva clathrata*. Regarding the globulin level, the results in our study showed an increase similar to the results obtained by Hoseinifar et al. [17] by adding 1% of *Gracilaria* to zebrafish feed.

Antioxidant enzymes such as SOD, CAT, and GPx are considered to be the first line of defence against the harmful effects of free radicals, which can be produced for various reasons and cause oxidative damage in body tissues [3, 55]. In the present research, it was observed that the addition of *U. intestinalis* to zebrafish diets caused an increase in SOD and GPx enzyme activities but did not cause a change in CAT enzyme activities. The results are in agreement with previous studies using various fish and shrimps and different macroalgae as feed additives. Akbary and Aminikhoei [46] in mullet fish showed that the best results in antioxidant enzyme activities such as superoxide dismutase, glutathione, and malondialdehyde in mullet, excluding CAT activity, were obtained from the water-soluble polysaccharide extract from the green algae *Ulva rigida* diet. Moreover, Liu et al. [24] showed that when they used polysaccharides from *U. intestinalis* (1 g kg^−1^) as feed additives, they effectively increased the activities of antioxidant enzymes in the hemolymph of *Fenneropenaeus merguiensis*, including total antioxidative capacity (T-AOC), SOD, GPx, and glutathione S-transferase (GST). It has also been shown that dichloromethane solvent extracts of *U. intestinalis* [16] and methanol extracts [56] showed good antioxidant activity in vitro conditions.

In contrast, unlike the present study, Guerreiro et al. [12] reported that there was no change in SOD and GPx antioxidant enzyme activities in the liver when they added *Chondrus crispus* and *Ulva lactuca* separately and as a mixture to their sea bream feed. Similarly, Peixoto reported that 7.5% *Gracilaria* sp. or an equal amount of 7.5% algae mixture (*Gracilaria* spp., *Ulva* spp., and *Fucus* spp.) added to the sea bass feed did not cause a change in antioxidant enzyme activities. In addition, Peasura et al. [57] showed that the addition of *U. intestinalis* did not cause a significant difference in the antioxidant enzyme activities of total glutathione (GT), glutathione peroxidase (GPx), and oxidised glutathione (GSSG) in the livers of sea bass.

In CAT activity, Peasura et al. [57], Peixoto et al. [51], Guerreiro et al. [12], Pezeshk et al. [11], and Akbary and Aminikhoei [46], who added *U. rigida* to mullet feeds, reported that it did not change in line with the current study. In contrast, Zhou et al. [47] reported that the polysaccharide obtained from *Enteromorpha prolifera*, which is one of the green algae, caused an increase in CAT antioxidant enzyme activity in the crucian carp *Carassius auratus* serum compared to the control group.

On the other hand, SOD antioxidant activities of fish fed with diets supplemented with macroalgae were also inconsistent with our results in some studies [11, 15]. Safavi et al. [15] showed that SOD activity in the liver of rainbow trout fed with 1.5 g kg^−1^ sulfated polysaccharides extracted from *Gracilariopsis persica* (SPG) and 0.5 g kg^−1^ sulfated polysaccharides extracted from *U. intestinalis* (SPU) for 8 weeks was significantly lower when compared with control group. Pezeshk et al. [11], on the other hand, reported that SOD activity was significantly reduced in *U. intestinalis* supplemented diets (*Labidochromis caeruleus*) compared to the control, in contrast to our study. The antioxidant capacity of macroalgae is attributed to the presence of antioxidant compounds such as carotenoids, certain polysaccharides, and polyphenols with scavenging activity, and they can neutralise these reactive oxygen species through their own oxidation due to their very high affinity for oxidative compounds [58]. Although most of the researchers suggested that there is a relationship between total phenolic content and antioxidant activity [40], some researchers claimed that they did not observe such a relationship [59, 60]. Ak and Turker [59], in their study with 5 macroalgae, reported that although they obtained the highest total phenolic content from *Cystoseira barbata* and the highest flavonoid activity from *Enteromorpha intestinalis*, they obtained the highest antioxidant activity from *Scytosiphon lomentaria*. It will be useful to carry out studies to shed light on this issue in future studies.

In the present study beside the levels of antioxidant enzymes activity, we checked the expression of antioxidant-related gene expression. This was performed to see if any upregulations occurred and either this upregulation is in line with elevation of enzyme activity. The inclusion of *U. intestinalis* in the zebrafish diets remarkably upregulated the SOD and CAT antioxidant-related gene expressions compared to control group. Similar results were observed in *Labeo rohita* fed with ulvan-containing diets [49] and in the hemocytes and gills of Pacific white shrimp (*Litopenaeus vannamei*) fed diet with hot water crude extract (HWCE) from *U. intestinalis* [10]. Also, Harikrishnan et al. [49] showed in their study that the antioxidant-related gene expressions such as SOD and GPx were remarkably upregulated in both challenged with *F. columnaris* and unchallenged groups fed with all ulvan supplementing diets (0, 25, 50, and 100 mg kg^−1^), except in challenged fish fed with 100 mg kg^−1^ ulvan diet when compared to control. Klongklaew reported that after the 21-day study, shrimp fed diets with 1 and 10 g kg^−1^*U. intestinalis* hot water crude extract (Ui-HWCE) showed higher expression levels than those fed diets supplemented with control and 5 g kg^−1^ Ui-HWCE. However, after 28 days, it was stated that all Ui-HWCE treatment groups (1, 5, and 10 g kg^−1^) exhibited significant SOD upregulation in the hemocytes compared to the control group (*P* < 0.05).

Molecular tools are increasingly used to evaluate the effects of various stress factors or nutrients on immunity, antioxidant, or growth in organisms at the molecular level [61–63]. In the present study, a significant amount of upregulation was detected in immune-related genes such as IL-1*β* (0.5-1%) and lyz (1%) in zebrafish fed with *U. intestinalis* added feed (Figure 1). IL-1*β* is an important cytokine involved in various cellular activities, including proliferation of T and B lymphocytes, and in the regulation of immune responses [57]. In the same context, Harikrishnan et al. [49] suggested that the expressions of IL-1*β*, lyz, and hepcidin cytokine genes were significantly upregulated in Labeo fish fed with ulvan enriched diet. In parallel with the findings of our study, Liu et al. [24] reported that when banana shrimp (*F. merguiensis*) were fed Enteromorpha polysaccharides additive diets at different rates for 42 days, lyz gene expression levels in the hepatopancreas, intestine, and gills were higher than the control group. However, in a recent study, Klongklaew et al. [10] reported that an upregulation of lyz gene expression was observed in in the hemocytes of Pacific white shrimp (*L. vannamei*) supplemented with 1 g kg^−1^ diet hot water crude extract from *U. intestinalis* (Ui-HWCE) after 28 days of feeding.

Seaweed has become a widely used method in fish diets, both as an immunostimulant and as a growth promoter [15]. Growth, which is coordinated by the GH-IGF system, is also affected by environmental factors such as temperature, photoperiod, and food availability [64, 65]. Since the growth rate of fish is a reflection of productivity and profitability in aquaculture, determining the effect of environmental and nutritional conditions on GH and IGF-I gene expression has significant potential in optimising fish health [66] and production [65, 67]. Therefore, it has been suggested that the GH-IGF-1 axis can be used as an indicator of growth performance and nutritional status in aquaculture [67]. Dietary *U. intestinalis* supplementation significantly increased the expression of growth-related genes (GH, IGF-1). Although, there is no report on the effects of dietary *U. intestinalis* on fish growth performance and related gene expression, it has been reported that green seaweeds (*Ulva* sp.) could improve growth performance in Nile tilapia [68]. Similar results have been reported by Mustafa and Nakagawa [69] in case of using small amount of seaweed in diet. Although there is no information available on the mode of action, Yone et al. [70] suggested that positive effect on growth performance can be due to an acceleration of nutrient absorption. Also, Nakagawa and Montgomery [71] stated that the seaweed's lipids comprise a wide range of fatty acids, including long-chain polyunsaturated important to neural function.

## 5. Conclusion

Green algae are renewable products with rich bioactive content that can be used as crude powder or by extracting them. In the current study, *U. intestinalis* was added to feed as crude powder and it was shown that it can be used as a feed additive. The best results achieved in 1% inclusion treatment. However, in case of several parameters no peak value appeared. It can be concluded that the beneficial effect may continue to increase when the amount of gutweed powder added to the feed is further increased. It may therefore make sense to control higher inclusion levels to get the best dose. However, the mechanism of beneficial results obtained using crude macroalgae powder was not investigated in the present study. In future studies, the effects of adding high levels as a feed component on digestive enzyme activities and intestinal morphology in cultured fish should be investigated. In addition, since the mechanism of action in fish has not been fully elucidated, studies should be implemented to determine the bioactive ingredients and then to determine the optimal dose of both the raw powders and the extracted bioactive components.

## Figures and Tables

**Table 1:** Chemical composition of the feed used as basal diet during the experiment.

Proximate analysis	(%)
Dry matter	93.6
Crude protein	38.9
Crude lipid	15.0
Ash	11

**Table 2:** Sequence and melting temperature (Tm) primers of selected gene expression related to mucosal immunity, antioxidant, and growth in zebrafish [43].

Primer name	Primer sequence	Tm	Application	Accession number
IL-1 q-PCRF	CGTCTCCACATCTCGTACTCA	58	Immune	AY340959.1
IL-1 q-PCRR	GTGTCTTTCCTGTCCATCTCC			
Lyz q-PCRF	GGCAGTGGTGTTTTTGTGTC	58	Immune	AY340959.1
Lyz q-PCRR	CGTAGTCCTTCCCCGTATCA			
SOD q-PCRF	GGGTGGCAATGAGGAAAG	58	Antioxidant	NM_139180.1
SOD q-PCRR	GCCCACATAGAAATGCACAG			
CAT q-PCRF	GCATGTTGGAAAGACGACAC	58	Antioxidant	AF210640.1
CAT q-PCRR	GTGGATGAAAGACGGAGACA			
IGF1 q-PCRF	AGTGTACCATGCGCTGTCTC	58	Growth	NM_131825.2
IGF1 q-PCRR	AATAAAAGCCCCTGTCTCCA			
GH q-PCRF	TTGGTGGTGGTTAGTTTGCT	58	Growth	AJ937858.1
GH q-PCRR	CTCAACTGTCTGCGTTCCTC			
*β*-Actin q-PCRF	AGCAGATGTGGATCAGCAAG	58	Housekeeping gene	NM_131031.1
*β*-Actin q-PCRR	TACCTCCCTTTGCCAGTTTC			

**Table 3:** The effects of zebrafish fed diets containing different levels of *U. intestinalis* on immune parameters in skin mucus and WBE.

	Control	0.25%	0.5%	1%
Mucosal total protein (mg/ml)	0.61 ± 0.1^b^	0.54 ± 0.15^ab^	0.77 ± 0.11^ab^	0.87 ± 0.15^a^
Mucosal immunoglobulin (mg/ml)	0.17 ± 0.07^b^	0.23 ± 0.04^ab^	0.31 ± 0.04^ab^	0.35 ± 0.05^a^
Mucosal lysozyme (U/ml)	7.53 ± 0.075^b^	7.96 ± 0.68^b^	8.43 ± 0.6^ab^	9.4 ± 0.7^a^
WBE total protein (mg/ml)	1.82 ± 0.28^b^	2.26 ± 0.17^a^	2.44 ± 0.22^a^	2.42 ± 0.22^a^
WBE immunoglobulin (mg/ml)	0.72 ± 0.07^b^	1.16 ± 0.16^a^	1.44 ± 0.18^a^	1.33 ± 0.14^a^
WBE lysozyme (U/ml)	18.43 ± 0.95^b^	21.23 ± 0.35^a^	21.57 ± 0.45^a^	21.99 ± 0.2^a^

Different letters on the same line indicate a significant difference (*P* < 0.05). Values are presented as the mean ± S.D.

**Table 4:** The effects of different levels of *U. intestinalis* on antioxidant defence of zebrafish.

	Control	0.25%	0.5%	1%
SOD (U/ml)	1870.46 ± 20.01^c^	1947 ± 43.92^c^	2621 ± 13^b^	2759.63 ± 17.97^a^
CAT (U/ml)	0.25 ± 0.02^a^	0.27 ± 0.04^a^	0.27 ± 0.04^a^	0.3 ± 0.04^a^
GPx (U/ml)	129.8 ± 9.6^b^	133.43 ± 6.27^b^	144.73 ± 12.83^ab^	156.56 ± 6.52^a^

Different letters on the same line indicate a significant difference (*P* < 0.05). Values are presented as the mean ± S.D.

## Data Availability

The data that support the findings of this study are available upon reasonable request to the corresponding authors.
